# Meaning of Gaze Behaviors in Individuals' Perception and Interpretation of Commercial Interior Environments: An Experimental Phenomenology Approach Involving Eye-Tracking

**DOI:** 10.3389/fpsyg.2021.581918

**Published:** 2021-08-16

**Authors:** Jain Kwon, Ju Yeon Kim

**Affiliations:** ^1^Interior Architecture and Design, Department of Design and Merchandising, Colorado State University, Fort Collins, CO, United States; ^2^Interior Architectural Design, School of Architecture, Soongsil University, Seoul, South Korea

**Keywords:** experimental phenomenology, eye-tracking (ET), interior design (ID), multisensory experience, spatial perception and cognition, visual attention (va), gaze behavior, commercial environment

## Abstract

A critical question in interior design is how multisensory information is integrated into occupant perception and interpretation of the environmental contexts and meanings. Although there have been efforts to identify and theorize visual perception of interior factors or features (e.g., colors, fixtures, and signs), the hidden meanings behind visual attention and behaviors have been neglected in interior design research. This experimental phenomenological study investigates the impact of auditory stimuli on the gaze behaviors of individuals and the hidden meanings of their audio-visual perceptions of commercial interiors. Implementing eye-tracking and open-ended interviews, this study explored how the neurophysiological and phenomenological methods in complementary can serve for interior design research on the meaning of gaze behaviors. The study used a convenience sample of 26 participants, three coffee shop interior images, and two musical stimuli. Essential to this study is the interpretive analysis of corresponding eye-tracking and interview data. The results show that visual perception is affected by auditory stimuli and other interior elements and factors associated with personal experiences; however, no distinct gaze pattern is identified by the type of auditory stimuli. The fixation patterns showed mixed reflections of the participants' perceptions, e.g., a single fixation pattern reflecting participants' likes and dislikes. Findings included six essential meanings of participants' gaze behaviors. This study suggested that auditory and visual stimuli are reciprocal in individuals' perceptions. Rather than one affects the other, the interaction between sensory stimuli contributes to the complexity and intensity of multisensory stimuli people associate with their experiences and conceptualize with meanings they establish.

## Introduction and Background

Over the past two decades, neurodesign and neuromarketing have become growing disciplines of study. Design research has increasingly given attention to the complexity and multimodality of sensation, perception, and cognition. The scope of research on perception and the applications of results have been broadened and diversified in various fields such as cognitive neuroscience, computer science, design, marketing, and psychophysiology. The conceptual stances concerning what constitutes perception vary. In certain disciplines, including psychophysics and psychophysiology, perception has been conceived as subjective and representative of the mind, while physical stimuli are considered objective and representative of the body (Hoffman, 2013). Crucial to the interior design inquiries and practice is the phenomenological translation of the covert essence of one's action and perception into architectonic dimensions, not only to identify individual elements seen on the surface; human perception and cognition are often approached in conjunction with individuals' conceptions of places and their associations. Spatial experience consists of perceptions of tangible (or reified) elements of a setting and also the intangible, such as the “atmosphere” or “energy.” Presently, various methods associated with emerging technologies [e.g., electroencephalography (EEG), eye-tracking, and virtual reality] play significant roles in design research concerning perception. The traditional design approach to understanding human perception has focused on the vision as the dominant human sense for acquiring information from the external environment. However, more recent design research presupposes that visual perception is part of spatial experience associated with emotions and feelings triggered by environmental attributes (Kwon, 2010, 2016; Lisińska-Kuśnierz and Krupa, 2020).

Due to the direct relationships among perceptions, emotions, and feelings, researchers have strived to measure complex human experiences in built environments properly. While emotions are immediate responses and reactions through biologically-based processes, feelings or moods persist as states span over more extended periods. Emotions and feelings play an important role in the behaviors and decision-making in built environments of individuals (Patel, 2005; Ibrahim, 2019); thus, these are critical factors to investigate in design research and how emotions and feelings are triggered. Emotions are how our brains respond to various stimuli and “tag” the information (e.g., positive or negative; relative intensity). Emotions are measurable; for example, they can be measured as to how “intense” or positive or negative—as shown in the circumplex model of affect (Posner et al., 2005), representing the psychological constructionist stance on measures of human emotions. In design research and consumer behavior studies, emotional responses have often been measured using self-reports (e.g., surveys and interviews), which can be useful when studies focus on whether some information (external stimuli) is memorable (Petermans et al., 2009; Umbas, 2015). Biometric measures of emotional responses have been increasingly implemented in interior design research, including using EEG to understand whether stimuli motivate the reactions of individuals and employing eye-tracking to study visual behaviors, such as what is noticed (Kalantari, 2019; Lisińska-Kuśnierz and Krupa, 2020).

Visual behaviors have been studied in cognitive neuroscience, computer science, consumer science, environmental design, and psychophysiology (Epelboim and Suppes, 2001; Andrá et al., 2015; Muldner and Burleston, 2015). Although eye-tracking is not a direct measure of visual acuity, it has been used as a useful tool in research and applications, such as usability research (Manhartsberger and Zellhofer, 2005), human factors research, and safety applications (Han et al., 2020), psychological/cognitive research (Mele and Federici, 2012), education and training (Tien et al., 2014), kinesiology and sports sciences (Lim et al., 2018), and car/airplane simulations (Palinko et al., 2010). However, a generally agreed limitation with eye-tracking data is that while these data can explain overt attention, i.e., what is observed and perceived, they do not contribute to understanding the covert, such as why and how. The limitation of eye-tracking and behavioral observation become significant shortcomings for design research, especially studies on occupant- or user-experience, e.g., post-occupancy evaluation. Due to these limitations, open-ended interviews and qualitative surveys have been used in design studies on human perception. However, the qualitative methods have often been criticized as lacking objectivity and limited in generalizability (Hegelund, 2005; Thorne, 2008; Charmaz, 2009; Cope, 2014; Vasileiou, 2018). Alternatively, studies justified qualitative and interpretive approaches for the richness of data, qualities of in-depth investigation, and the value of interpretive phenomenological analysis using a small sample (Braun and Clarke, 2006; Smith et al., 2009).

Our understanding of spatial perception and cognition remains limited due to the complexity of measuring, analyzing, and interpreting the phenomena. Auditory and visual stimuli always exist in ordinary interior settings, while the other sensory input can be more specific (to a certain degree) to their occupancy types, e.g., smell and taste foods at restaurants and touch clothes at garment stores. Individuals' attention to various visual information is requisite to their decision-making on purchasing in commercial environments. Although online shopping has become prevalent in the retail market of today, places like coffee shops inevitably involve physical settings to some degree—even for an online order pick-up—comprised of the human–human and human–environment contact and interaction in the multisensory environment. By implementing mixed methods that involved eye-tracking and qualitative interviews, this study explored an experimental phenomenological approach to investigate the relationship between individuals' gaze behaviors and the covert dimension of their audio-visual perceptions of commercial interior settings. This study uses coffee shop images as a convenient example of ordinary commercial interiors. Underpinning the multisensory concept of this study focused on the audio-visual is the phenomenology of perception of Merleau-Ponty (Merleau-Ponty and Landes, 2012): the senses are distinct yet indiscernible as united through the body in becoming its perception (2014). Based on the notion, this study presupposes that audio and visual perceptions are interwoven in a holistic multisensory experience.

The relationship and differences between philosophical phenomenology and experimental science have been discussed and interpreted such that science can explain what is observed and perceived, while phenomenology can provide information about why and how. Thus, qualitative-descriptive or phenomenological analysis and neurophysiological analysis are complementary, not contradictory (Vicario, 1993; Ihde, 2012; Albertazzi, 2019). One of the underlying assumptions of experimental phenomenology is that qualitative phenomena are irreducible to stimuli (Albertazzi, 2013). Experimental phenomenology includes empirical and theoretical approaches in juxtaposition. Although some might view the assertion as paradoxical, the logic in it is that researchers attempt not only to identify “what” they investigate (e.g., Minors and Harvey, 2015) but also uncover “how” and “why.” Phenomenology is concerned with individuals' lived experiences and perceptions. Phenomenologists assert that human experiences are lived and subjective, the essences of which are not reducible to stimuli; moreover, they argue that neurophysiological data do not have the explanatory capacity (Albertazzi, 2013). Criticisms on phenomenology are that its methods and findings are unclear and limited and do not provide outcomes with pragmatic solutions for practicing professionals who often have more immediate goals than searching for the essential (Oliver, 2012, p. 410). From a phenomenological point of view, physiological and neuroscientific methods are too analytical to adequately explore the essences of human experiences, including visual perceptions (Vicario, 1993). As human perception is multifaceted, it may reveal unknown truth when approached from multiple points of view. Along with its experimental approach to gaze behaviors, this study implements phenomenological analysis, “a probing of what is genuinely discoverable and potentially there, but not often seen” (Ihde, 2012, p. 13).

Adopting an experimental phenomenological approach, this study investigates how auditory stimuli affect gaze behaviors and the hidden meanings behind the gaze patterns in audio-visual settings in commercial contexts. This study presupposes hidden meanings behind gaze behaviors, e.g., why individuals notice, pay attention to, and remember certain spatial attributes or elements; qualitative measures are requisite for uncovering the meanings that cannot be predicted through quantitative approaches.

The research questions of the study are:

1) What are the hidden meanings behind individuals' gaze behaviors in their audio-visual perception of interior settings?
2) Are gaze behaviors affected by the types of musical stimuli in coffee shops?
3) Do gaze behaviors represent visual preferences of interior settings?

This study implements mixed methods that involve lab-based eye-tracking and an open-ended interview. As the researchers did not find field-specific precedents involving qualitative and quantitative measures, this study gives greater attention to uncovering the essential meanings hidden behind gaze behaviors than testing hypotheses that may conflict with the phenomenological aspect integrated into this study.

## Eye-Tracking in Design and Retail Research

Interior design aimed to provide human environments through the creative process balanced with a critical point of view on occupant needs and desires that are often subjective. Due to this two-fold approach, no single form of inquiry may sufficiently examine human experience in built environments. Instead, integrated approaches may better understand the complex nature of and interactions among the human senses and how a multisensory context affects one's perception of space. Spatial experiences involve complex and multimodal perception and cognition. Studies have found that the human brain can learn and process cue associations through multisensory experiences such as audio-visual and tactile-visual (Gori et al., 2012; Wismeijer et al., 2012). Cue associations are not innately present but established through individuals' subjective experiences that affect the perception of the individual and interpretations of the context. Minors and Harvey (2015) examined how the visual aspects of building interiors impact the acoustical experience of an audience in a concert hall. They pointed out that, when designing contemporary buildings, significantly less attention and time are allocated to finish details; instead, the focus is on building form. They suggested that gaining insight into what people look at may help designers understand what to focus their efforts on in design processes. Audio-visual experiences have been studied in consumer research. Mehta et al. (2012) examined how ambient noise in a cafeteria can affect creativity using a creativity test tool. They found that “a moderate level (70 dB) of ambient noise enhances performance on creative tasks and “increases the buying likelihood of innovative products” (p. 785). However, because ambient noise can vary, this finding may not be applied to all interior environments.

Eye-tracking has been used in design studies on spatial identification, navigation, and wayfinding (Viaene et al., 2016; Tang and Auffrey, 2018; Su et al., 2021). Eye-tracking is a useful method to measure immediate gaze responses to visual stimuli; the cognitive process, including the emotional or conscious motives or triggers for eye-fixations on specific objects of the individual, can be sought through phenomenological measures, including interviews. Eye-tracking research has its historical roots in cognitive research on reading (Just and Carpenter, 1976). In the early research and some recent studies on visual attention, the eye-mind hypothesis (Just and Carpenter, 1980) seemed to be adopted as a strong validation for the power of eye-tracking. The essence of the hypothesis is that people tend to pay attention to and think about what they are seeing. However, in some cases, the hypothesis might lead to overgeneralization or oversimplification, undermining the qualitative aspects of perception because mental processes are not always aligned with visual attention (Schindler and Lilienthal, 2019). Eye-tracking has been used in studies on visual behaviors of people in built environments, e.g., how individuals visually navigate space and orient themselves in environmental settings (Mazman and Altun, 2013; Viaene et al., 2016; Guntarik et al., 2018); how eye movement responds to other sensory input in wind parks (Yu et al., 2017). Cognitive studies on esthetic judgments argued that gaze fixations are affected by the symmetry in architecture, visual arts, and faces; visual behavior represents an aesthetic preference for visual configuration and balance (Treder, 2010; Hodgson, 2011; Giannouli, 2013). Eye-tracking has also been implemented in retail studies: (1) the visual attention of customers to signage and products affects their purchasing probability at retail stores (Huddleston et al., 2015; Tang and Auffrey, 2018); (2) directional patterns (e.g., vertical and horizontal sightlines) in the visual navigation of consumers on retail displays (Atalay and Meloy, 2011; Goldberg and Helfman, 2011; Deng et al., 2016); (3) there is no significant or direct effect of the first fixation on consumer choice (van Der Laan et al., 2015); (4) consumers have a more favorable attitude and positive perception toward merchandise and service quality and feel more aroused or pleased in a store with social cues presented, e.g., in-store displays of graphics with a social implication (Hu and Jasper, 2006).

Interior design research has adopted eye-tracking, as gaze data can help reveal—to a certain degree—unspoken thoughts and biases that often occur in self-reports. However, the covert side of visual behaviors, the deeper meaning of the association between the thoughts and gaze behaviors of individuals, has not been discussed. Interior design practitioners and researchers put efforts into understanding the feelings, preferences, and interpretations of occupants of interior environments, not merely identifying material objects or features that might catch the eyes of the people. This study investigates whether and how auditory stimuli affect the gaze behaviors of individuals and the hidden meanings behind their visual attention to spatial attributes of commercial interior settings, particularly coffee shops.

## Methods

### Eye-Tracking Metrics

Before detailing the research methods of this study, it seems necessary to overview the key terms and metrics of eye-tracking concerning a wider audience interested in adopting eye-tracking in research. The essential contents and metrics of eye-tracking data include gaze point, fixation, fixation duration, dwell time, and area of interest. These measures allow to verification of the visual patterns of individuals who have various levels of cognitive performance. A *gaze point* refers to the spot looked, which equals one raw data point captured by an eye tracker. For example, if the eye tracker measures 30 times a second (30 Hz), each gaze point represents a 13th of a second (or 33.33 ms). A cluster of gaze points in close proximity constitutes a *fixation* that is an effective measure of visual attention. *Fixation count* reveals how often a participant viewed the area of interest or refocused attention to that element (Huddleston et al., 2015, p. 568). The *time to first fixation* (TTFF) refers to the amount of time it takes an individual to look at a specific area of interest. The TTFF can indicate both bottom-up stimulus-driven searches (e.g., a neon sign catching immediate attention) and top-down attention-driven searches (e.g., individuals actively decide to search for specific elements or areas in a picture). *Areas of interest* (AOIs) are user-defined sub-regions of a displayed stimulus and are essential in analyzing eye movement and fixation data. The methods of AOI construction can vary and include hand-drawn (or selected) AOI, Voronoi tessellation, limited-radius Voronoi tessellation (LRVT), and grid methods. Types of AOIs include dynamic AOIs, gridded AOIs, planes, and whitespace. The type of AOIs is determined based on the subject of the study. Often in retail studies on consumers' visual attention to products and information on purchase intention, AOIs are created around products or price display signage in a specific display (Huddleston et al., 2018). *Dwell time* is the amount of time that a respondent spends looking at an AOI. Studies suggested that dwell time often indicates a motivational determinant and conscious attention. For example, longer dwell times, i.e., prolonged visual attention to a specific area, suggests a higher level of interest, while shorter dwell times indicate contents that might be more catchy (Farnsworth, 2018).

Heatmaps and scanpath plots (or gaze plots) are used to visualize gaze data. A *heatmap* displays the distributions of visual attention, which effectively reveals the focus of visual attention for a group of participants during the same time. In heatmaps, there is no information about the order of gaze points or focus on individual fixations. Heatmaps are color-coded, with red areas indicating higher fixation counts and suggesting a higher level of interest; yellow and green areas indicate lower fixation counts and lower levels of visual interest. Areas without coloring are likely to receive little or no attention. *Gaze plots* (or *scanpath plots*) show the location, order, and time spent looking at locations on the stimulus. The primary function of gaze plots is to reveal the sequence of where individuals look. Fixation duration refers to the time spent gazing and is shown by the diameter of the fixation circles in the data display (**Table 5** in 5.1.3), i.e., the longer the gazing time, the larger the circle.

### Sample

The sample size for this study was determined based on the recommendations and guidelines found in precedent studies. Creswell (2013) suggested that 20 to 30 interviews be adequate for qualitative analysis; Morse (2000) suggested 30 as a working number for semi-structured interviews to reach theoretical saturation. Marshall (2013) analyzed 83 studies from information systems journals and recommended 15–30 for single case projects; Vasileiou (2018) analyzed 214 health research articles and found the medians of the sample sizes used in the studies published in three leading journals ranged from 15 to 31.

This study used a voluntary sample of 21 (*N* = 21) that consisted of 14 female (*n* = 14) and seven male (*n* = 7) university students in various majors, i.e., accounting, communication, computer science, design, fine arts, journalism, management, and mechanical engineering. For design students, the eligibility to participate in the study was restricted to pre-major first-year. The eligibility for all participants was restricted to US residents and English-first-language speakers. The ages of the participant ranged from 19 to 23 years old (M = 20.9; SD = ±1.35). Because the study was conducted without comparing data between gender groups, the researchers did not attempt to balance the participants in equivalent numbers. The eligibility for research participation was limited to individuals with no vision impairment: naked or corrected binocular eyesight +0.5 or higher; no color deficiency. For color deficiency tests, *Ishihara's test for color deficiency* (Ishihara, 2012) was used.

### Equipment and Tools

The quantitative method used in this study was derived from a prior study on gaze fixations, saccades, and blinks in audio-visual perception (Kim and Kim, 2020). However, the study pointed out that its quantitative tool and method could neither the meaning of the gaze behaviors nor the “joint effect of multisensory cues” (p. 9). Regarding the critical limitations, this study aimed to explore the qualitative and interpretive facets of gaze behaviors in interior environments.

For unobtrusive head-free eye-tracking, a screen-mounted eye tracker, SMI REDn (with a sampling rate of 30 Hz), was integrated into a 27-in. 1,920 × 1,080 pixels widescreen monitor. SMI BeGaze 3.7 was used for analysis and visualization of raw gaze data. Normalizing light and controlling noise is crucial in eye-tracking because various environmental factors can cause blinks and lookaways and thus, affect the quality of eye-tracking measurement. Eye-tracking experiments were conducted in a lab setting that resembled a darkroom in a quiet location to isolate the experiment setting from sensory distractors.

**Figure 1:**
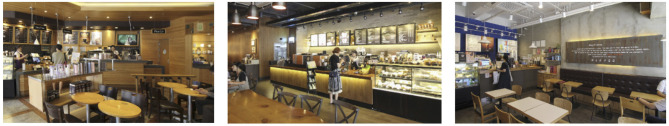
Visual stimuli for eye-tracking experiments—photos of three coffee shops, from left, I-1, I-2, and I-3 (Kwon and Kim, 2018, p. 82; 2020, p. 447).

The visual stimuli used were photo images of three franchised-brand coffee shops (Figure 1) located in Seoul, Korea, with no culture-specific design feature. Each image shows ~8 × 6.5 m of the interior space of a coffee shop, including the order/pick-up and seating areas. The viewing distance for eye-tracking was set at 600–650 mm. The images naturally include coffee shop workers and customers *in situ*. Each image shows four people (including full and partial figures), including one customer interacting with a worker. The researchers did not modify the visual presentation of the interior configuration, as the real-world situations were better suited to the study than ideally controlled mock-ups that better fit studies testing hypotheses.

As auditory stimuli, two songs in different music genres, jazz-pop (M1) and dance-pop (M2) were used. These songs were chosen to simulate two common types of sound atmospheres in commercial settings. A field survey was conducted to identify the most played musical genres in eighty coffee shops. The musical pieces played in the coffee shops were categorized into six genres (i.e., new age, dance-pop, ballade, old pop, electronic, and jazz-pop). Of the six, jazz-pop and dance-pop showed the highest frequency scores (*N* = 109: jazz-pop = 37; dance-pop = 29; ballade = 23; old pop = 14; no music = 3; electronic = 2; new-age = 1). A jazz-pop song (M1) in 88 beats per minute (BMP) and a dance-pop (M2) in 137 BPM were selected and used as the auditory stimuli in data collection. The languages used in lyrics were not controlled: M1 in English and M2 in Korean.

An open-ended interview questionnaire was used: primary questions (10) and up to three probing questions for each of the primary. Probing questions were selectively asked, dependent upon the responses of each participant to the primary questions. Because an interview is a self-report by its nature and is often in a face-to-face setting, interview participants sometimes seek “correct answers” when responding to questions, which results in overly articulated or summarized responses. To minimize such a risk, an open-ended interview questionnaire was carefully designed based on the construct of the symbolic interactionism of Blumer (1969): the self (self-conception/identification), object (abstract, physical, or social), social interaction, and joint action. The symbolic interaction framework is suited to this study, as its holistic view concerns the meaning of human–human and human–environment interactions, which is essential to research on spatial experience. The sequence of interview questions was derived from prior studies adopting symbolic interaction approaches to research on meanings of interior environments (Kwon, 2010, 2016). The questionnaire also included questions about the experience of participants of the eye-tracking experiment setup and procedures at the end.

### Data Collection

The eye-tracking data of each participant were collected through the following procedures:

1) Before showing images to each participant in eye-tracking, a short instruction was displayed on the monitor: “please look at the image on the screen as if you are looking around inside the coffee shop presented.”
2) One photo image paired with a song was displayed on the screen (viewing distance 600–650 mm) for one 60-s period.
3) A 10-s break was given between two sessions while the monitor displayed a blank white screen.
4) Per participant, the procedures (2) and (3) were repeated six times in total, using I-1, I-2, and I-3 paired with M1 and M2 in random order: I-1/M1, I-1/M2, I-2/M1, I-2/M2, I-3/M1, and I-3/M2.

A 30-min open-ended interview followed the eye-tracking experiment of each participant. Interviews were conducted in a separate meeting room adjacent to the eye-tracking lab, without sensory cues for retrospective recall. All interviews were conducted by the same interviewer who was not previously acquainted with the interviewees. The open-ended retrospective interviews focused on the feelings and thoughts of participants on the perceived spaces and sounds during their eye-tracking experiments. The participants were also asked a separate set of questions about their experience of the eye-tracking experiment set-up and procedures at the end of the interview.

### Data Analysis and Interpretation

Data analysis consisted of four phases:

- Quantitative analysis and visualization of gaze data
- Content analysis of the visualized gaze data
- Content analysis of interview responses
- Comparison of the three sets of analyzed data.

A total of 3,600 (60 s × 2 music pieces × 30 Hz) fixation data per participant was collected and analyzed. Each 60-s session associated with one music piece was broken into six 10-s segments (from T1–T6). The 10-s unit was determined based on precedent studies (Kim and Jung, 2012; Kim and Kim, 2018) on time-sequences in spatial observation, which compared 3-, 5-, 10-, 15-, and 30-s segments and found 10-s most effective. The averages of fixation count and fixation duration with music 1 (M1) and with music 2 (M2) were compared. Scanpath analysis was conducted to identify similar patterns of fixations among participants. In this study, the 12 × 12 Gridded AOIs (Table 1) were used. In Table 1, the number shown in each AOI grid indicates the average of the fixation count in the AOI. Heatmaps were generated by time-segment (10 s per segment) to visualize the general distribution of gaze points and identify the primary AOIs as the participants engage in the images.

**Table 1:** 12 × 12 gridded areas of interest (AOIs) of I-1, by music and time-segment (T1 exampled).

	**Jazz Pop (M1)**	**Dance Pop (M2)**
T1: 00-10 sec.	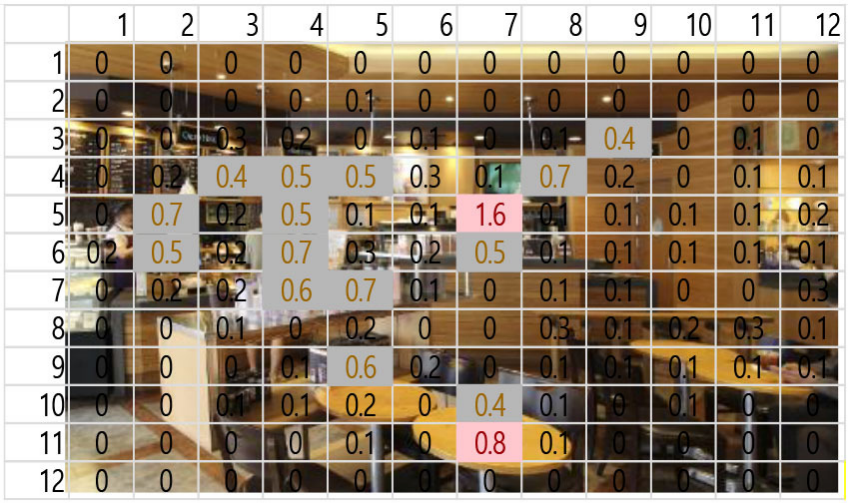	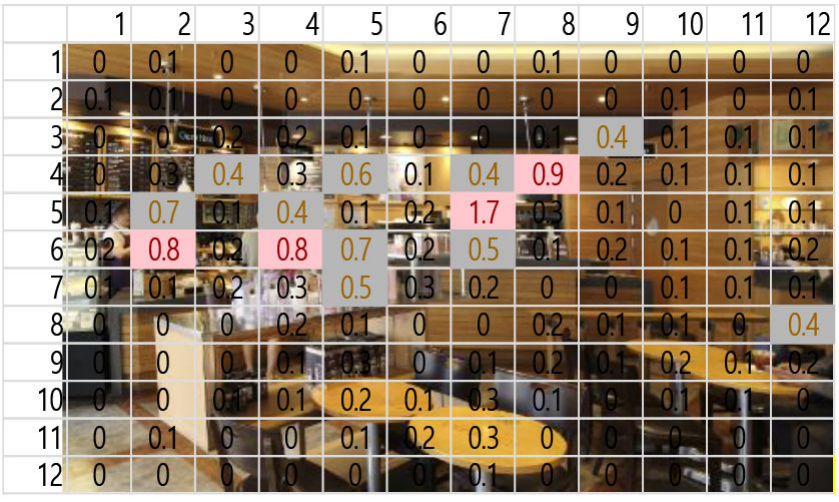

The interview analysis included both descriptive and interpretive phenomenological processes. All interview responses were transcribed for content analysis. Due to the highly descriptive and various wordings by the individual, the interview responses transcribed were categorized by thematic context responding to the construct of symbolic interaction: brand/commercial elements to *object*, interior factors/atmosphere (including sound) to *object*, personal memories/associations to *the self*, human interaction to *social interaction*, and cultural factors to *joint action*. The categorized responses were analyzed in-depth compared with corresponding gaze data; then, multiple themes were derived from each context category; the researchers synthesized and interpreted the themes and determined six essential themes. Although the symbolic interaction criteria were used to categorize interview responses in the initial phase of analysis, the researchers did not attempt to match the six essential themes found back with the criteria because each essential theme encompassed multiple criteria of symbolic interaction.

## Results

The fall-out rate was 4.7%: 20 (13 females and 7 males) of the total sample (*N* = 21) were considered valid and were analyzed. The results illustrated in this section include the participants' gaze patterns found through eye-tracking and the contents or determinants (the “why”) of the visual behaviors, which were found through in-depth interviews. Some limitations related to the findings from data analysis are also discussed in this section.

### Findings From Eye-Tracking

Overall, the collected gaze data revealed considerable visual attention to areas with a quantity and complexity of visual information, including signs, menu, and sales products. The general patterns of the participants' fixations and scanpaths differed depending on the type of auditory stimuli, i.e., the music played during the experiments. Besides the interior elements and objects, the participants tended to pay attention to the interactions of people they found in the images. The following are the details of the essential metrics measured in this study.

#### First Fixations

In this study, the first fixated objects/contents and the pattern among the individual participants' TTFFs were important. Participants' first fixations were analyzed by time and contents: first fixations on each AOI were counted, and the contents in the first fixated AOIs were analyzed. The highest numbers of first fixations on the contents in I-1 and I-2 appeared in the order: (1) human figures, (2) corners or edges of interior elements or objects, and (3) signs, menus, or decorative text. The same order was found when I-1 was displayed, regardless of the music played. In I-2, many of the first fixations appeared on the two persons in interaction near the center of the image regardless of the music. When I-3 was displayed, the first fixations appeared in the order of (1) the large decorative text—“something to figure out”—on the wall, (2) corners and edges, and (3) human figures with M2 played, which appeared in the reversed order when M1 was played. With M1, 40% (n=8) of the first fixations (FF) were on two of the three human figures in the image (seven on one facing the viewer); the third FF (0.21 s) was on one facing the viewer. With M2, 70% (*n* = 14) appeared on the two persons in the image (50% on one facing the viewer and 20% on one showing its back).

**Figure 2:**
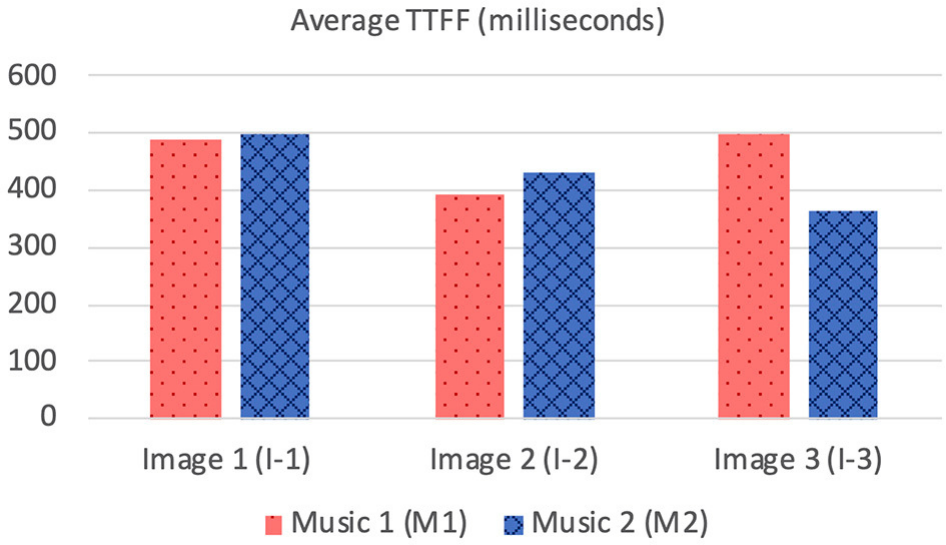
Average time to first fixations.

Three images (I-1, I-2, and I-3) showed the same pattern: the average time to first fixation (TTFF) was longer when M1 was playing (Figure 2). Individual participants' TTFFs were varied. In I-1, 50% (n = 10) of the participants showed longer TTFF with M1 and 40% (n = 8) with M2. Two participants showed no difference in TTFF between the two auditory stimuli. The eighth of the 14 FFs took the shortest time (0.39 s), which was fixated on one facing the viewer. On I-2, 60% (n=12) participants showed a longer TTFF with M1, 35% (6) with M2, and two participants showed no difference between the two auditory stimuli. Seven of the 12 with M1 played were significant, and seven of the seven were significant when M2 was played. In I-3, 35% (*n* = 7) of participants showed longer TTFF with M1, 60% (*n* = 12) with M2. One participant showed no difference between the two auditory stimuli.

Although the type of each auditory stimulus by itself did not directly affect participants' first fixations or immediate visual attention, their interview responses (detailed in Section Findings from interviews: meanings of gaze behaviors) implied their engagement in analytical processes in the early phase of their visual experience with the faster music (M2). For example, the early fixations on corners of the interior structures and objects were related to the participants' attempts to visually define objects and interior configurations.

#### Gaze Fixations and Visual Attention

Regardless of the music played, the ranges of the average fixation count (Table 2) and the mean dwell time (Table 3) by time segment were greater with I-2 than I-1 and I-3. Overall, the average fixation count was higher in T1 (0–10 s) than T6 (50–60 s), and the mean dwell time was lower in T1 than T6. All fixation counts and durations were analyzed to compare the time to gaze “concentration,” i.e., 300 ms and longer durations, and “high concentration,” i.e., the longest 10% of the fixation data (Table 4). Regardless of the stimuli used, the average time to gaze concentration appeared between 11 and 15 s in T2 and high concentration between 32 and 39 s in T4. The results did not show specific patterns of differences in the types of music played. The faster or complex sounds affect individuals' grasp of surroundings and concentration on visual perceptions. However, the result should be further scrutinized as the time to gaze concentration and high gaze concentration was variable for individuals.

**Table 2:** Average fixation count by time segment.

**Image 1 (I-1)**	**Image 2 (I-2)**	**Image 3 (I-3)**
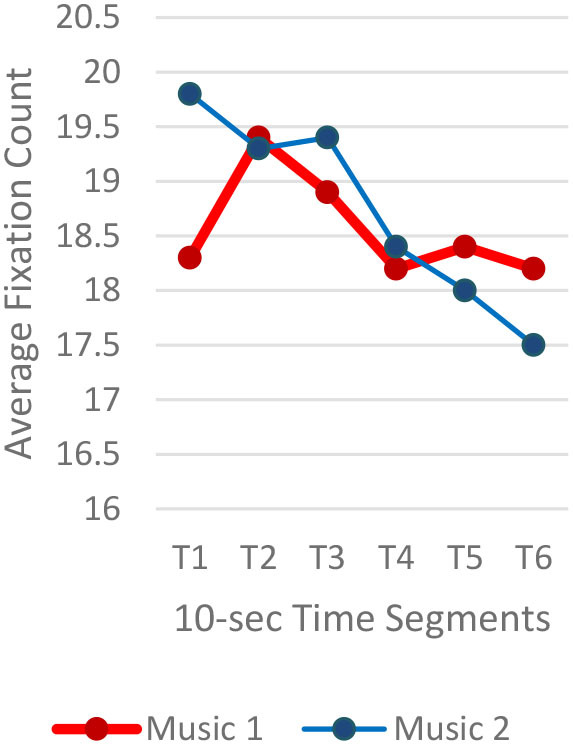	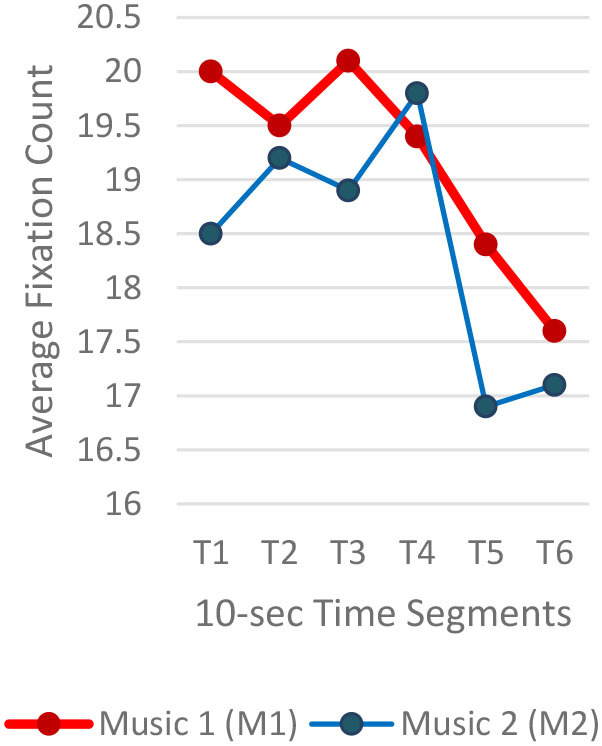	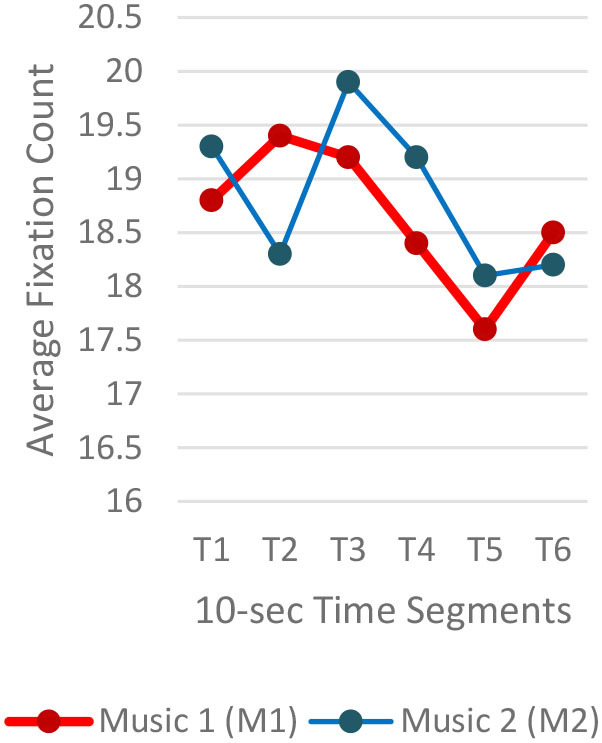

**Table 3:** Mean dwell time by time segment.

**Image 1 (I-1)**	**Image 2 (I-2)**	**Image 3 (I-3)**
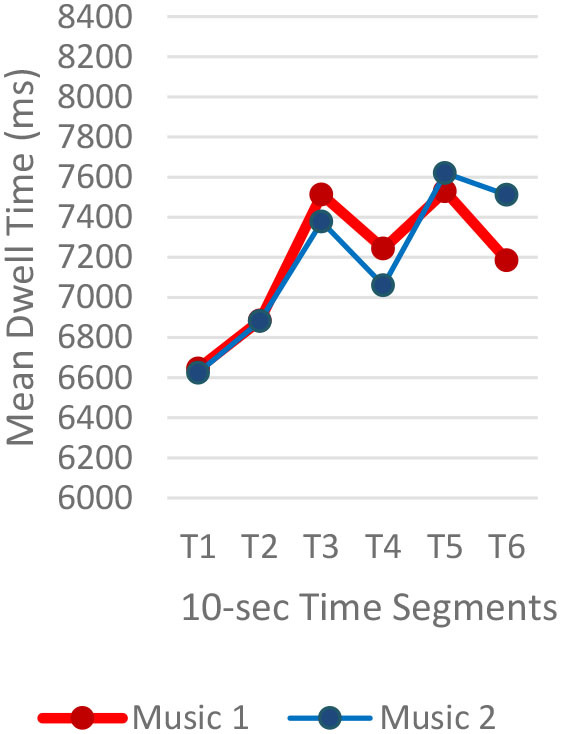	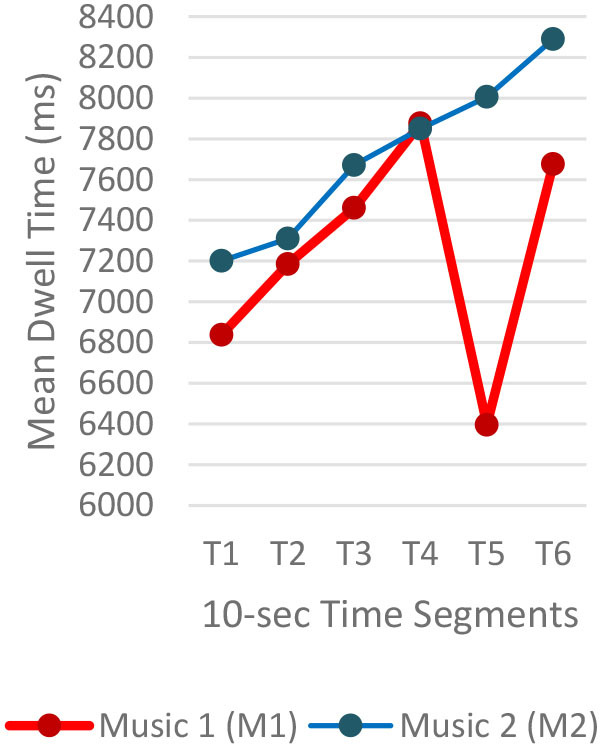	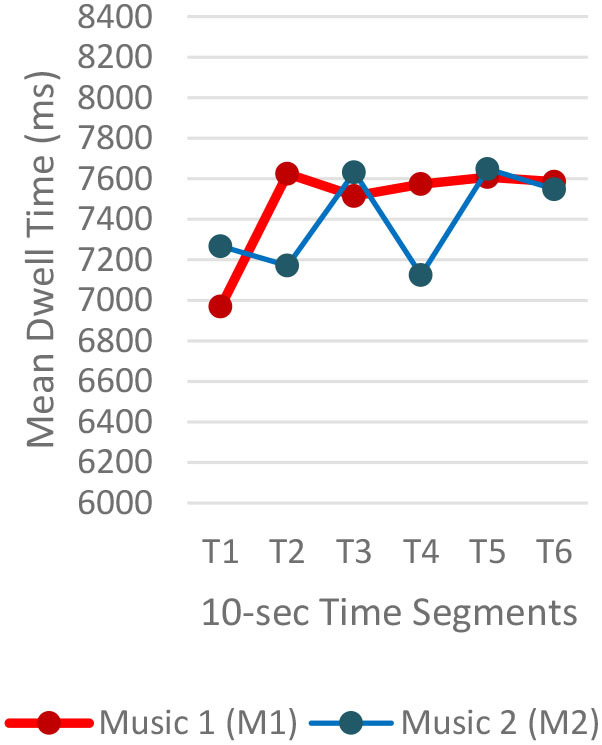

**Table 4:** Average time to concentration and high concentration.

**I-1**	**I-2**	**I-3**
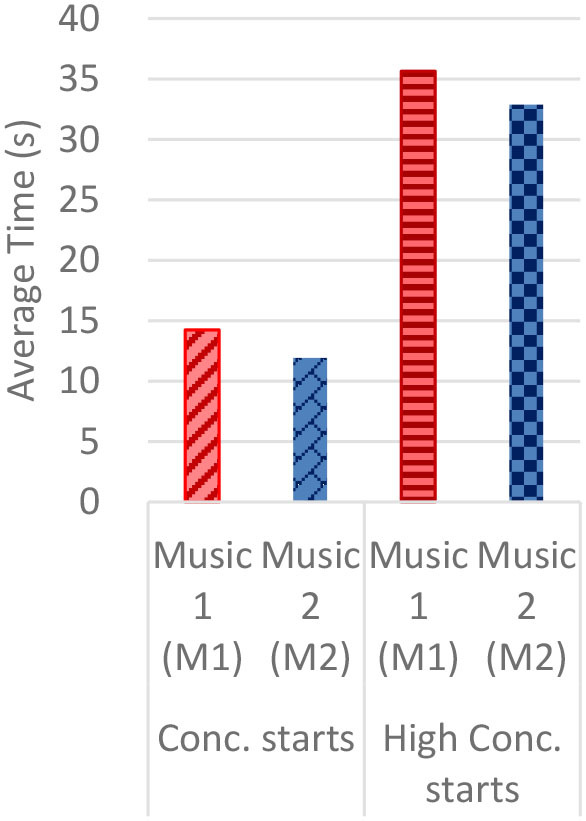	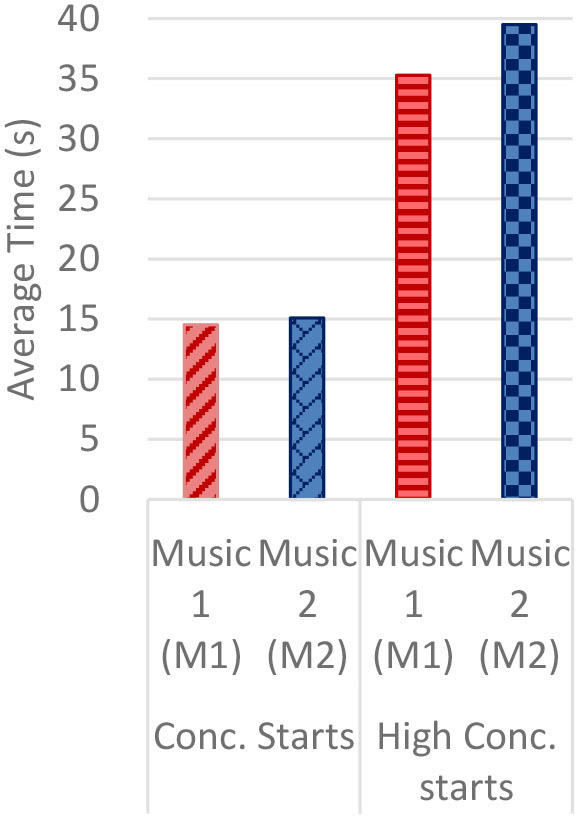	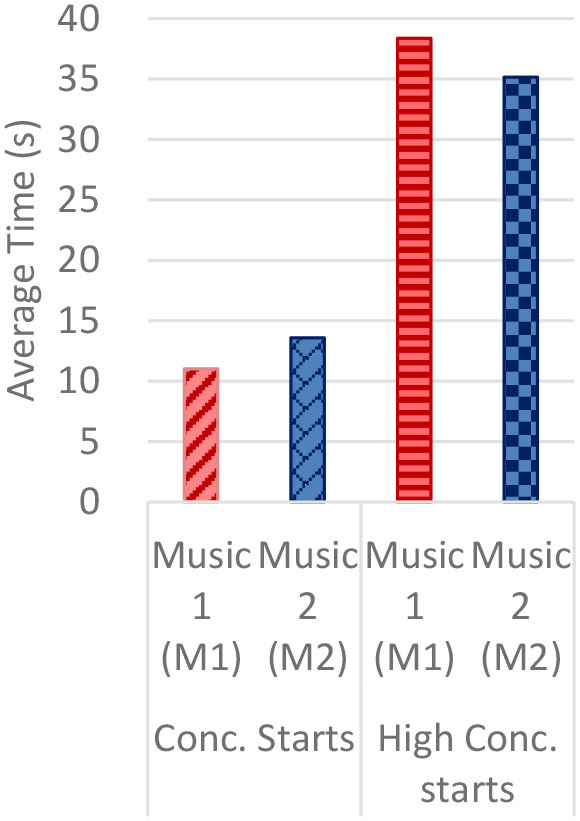

#### Gaze Plots and Heatmaps

When visualized, the gaze plots (Table 5) showed a more scattered pattern with M2, while higher fixation counts appeared in fewer AOIs with M1. Despite the differences in individual participants' scanpaths, general patterns were also found. With M1, there are more concentrated fixations, i.e., higher fixation counts and longer dwell time per AOI were found. With M2, more scattered fixations and shorter dwell times were found. This result reiterates the finding above, showing that fast or complex sounds may be a greater distractor and stressor, affecting individuals' grasp of surroundings and concentration on their visual perception. The findings from the open-ended interviews (see Section Eye-Tracking in Design and Retail Research) provide in-depth explanations of the various factors and attributes that trigger or affect such visual behaviors.

When no specific visual information (e.g., signs, menus, sales products, etc.) was provided, the architectonic configurations (wall corners, open ceilings, etc.) contributed to the participants' visual learning of the spatial order. In contrast to other studies using eye-tracking, it was unclear in this study whether the participants' visual attention tended to fall near the center of the images because of the visual symmetry. This result is also reflected in the heatmaps (Table 6), representing all participants considered valid (*n* = 20). Although a heatmap may show the general gaze pattern, the heatmap alone cannot provide sufficient content that designers often need, including the causes and influences on users' visual attention to specific areas or objects. Participants' interview responses detailed in the following Section (Findings from interviews: meanings of gaze behaviors) revealed more varied reasons for their gaze behaviors.

**Table 5:** Gaze plots by music (Participants #20, #09, and #05 exampled).

	**M1**	**M2**
I-1 (P20)	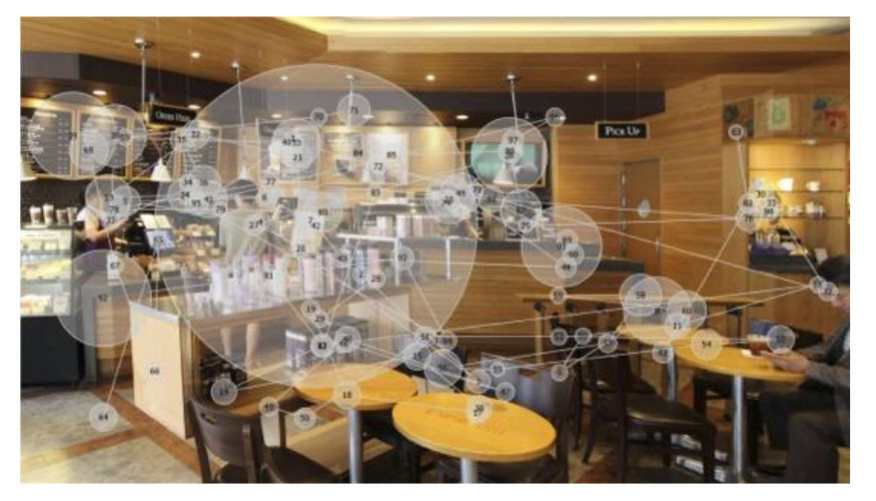	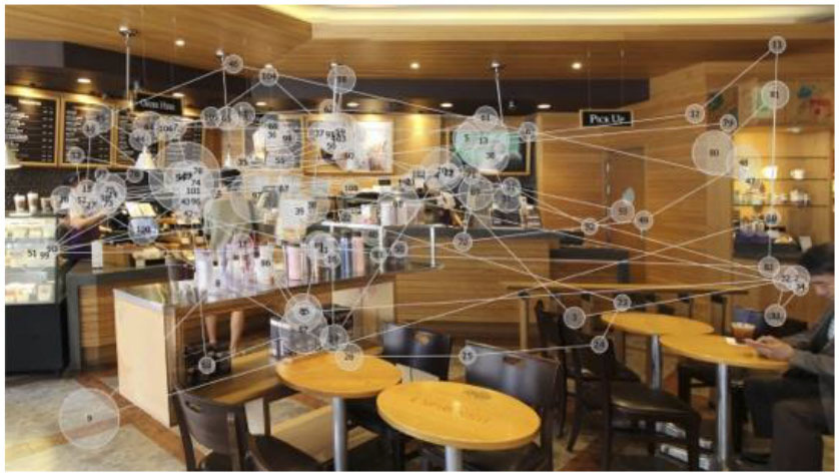
I-2 (P09)	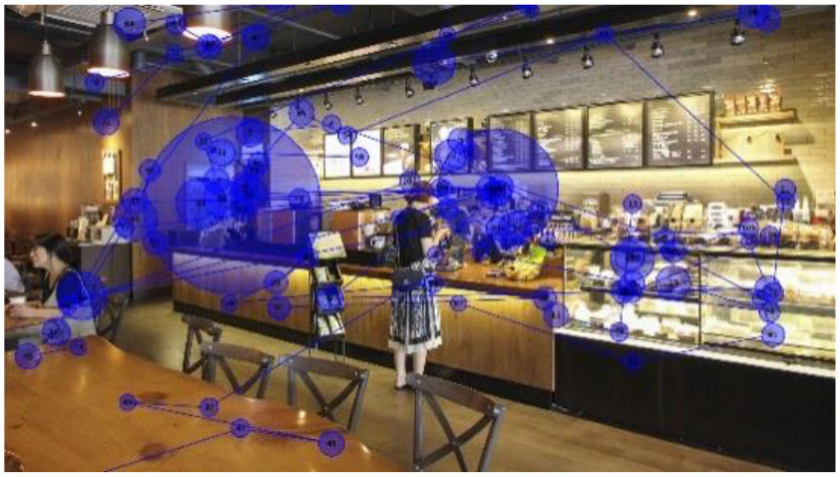	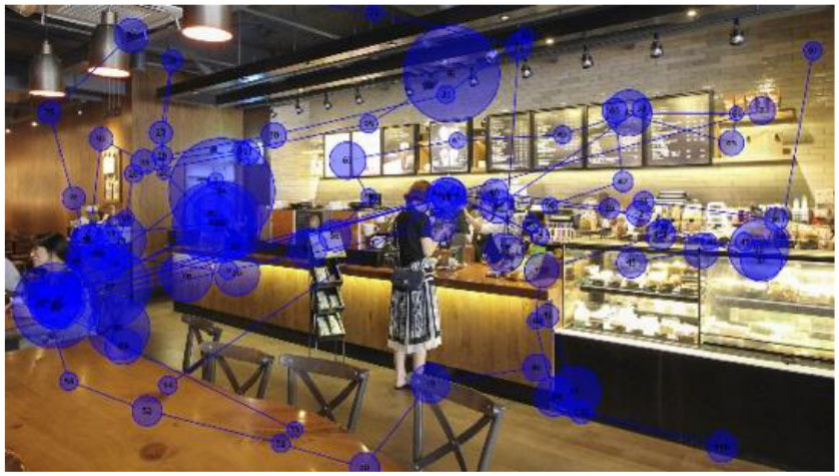
I-3 (P05)	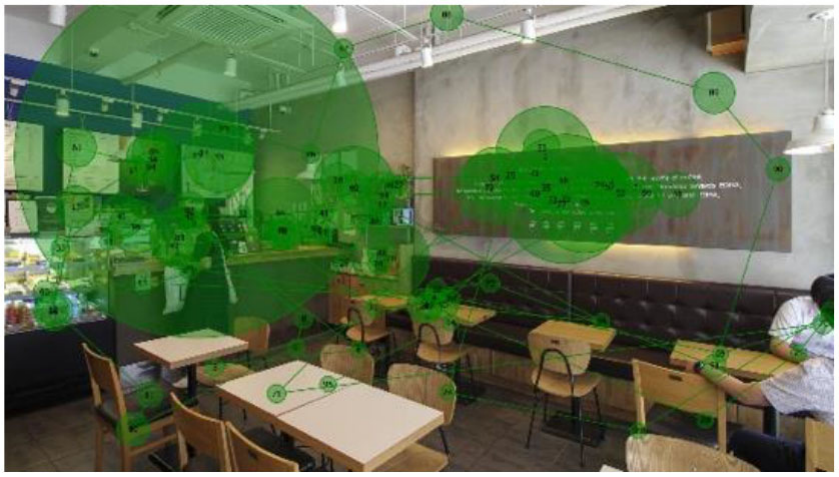	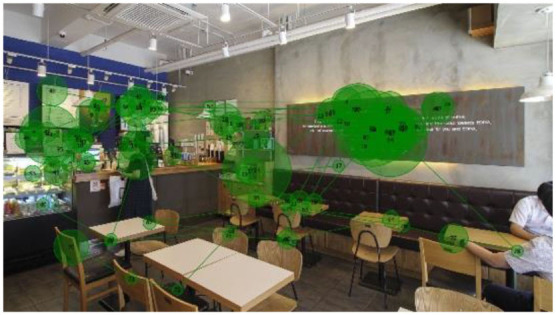

**Table 6:** Heatmaps: red areas indicate higher fixation counts and yellow and green areas show lower fixation counts.

	**M1 (jazz pop)**	**M2 (dance-pop)**
I-1 (T1-T6)	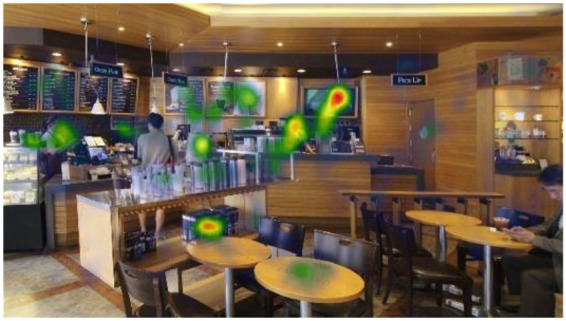	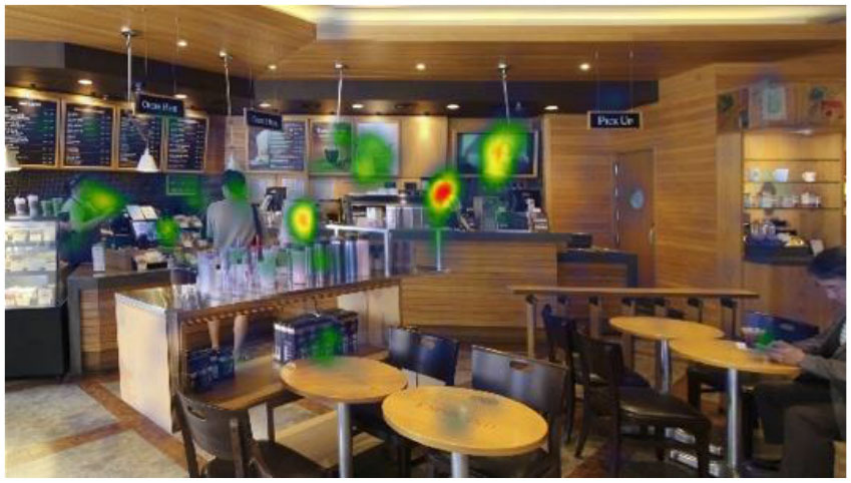
I-2 (T1-T6)	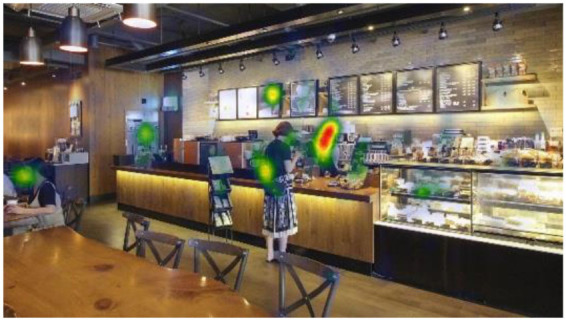	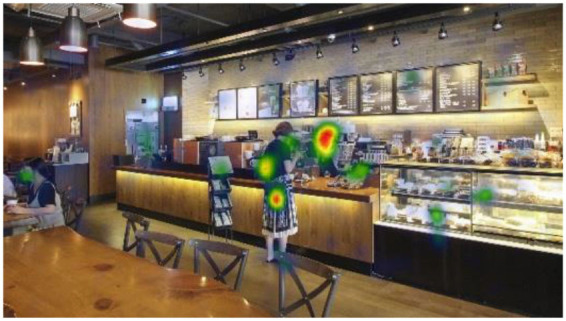
I-3 (T1-T6)	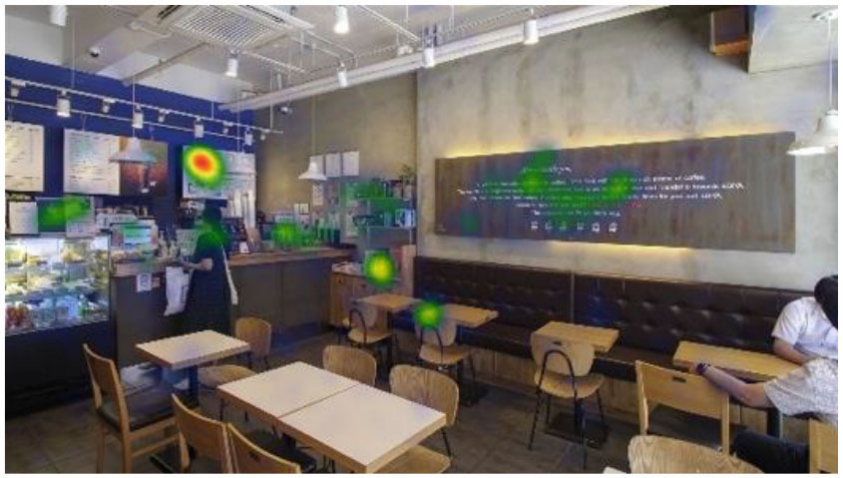	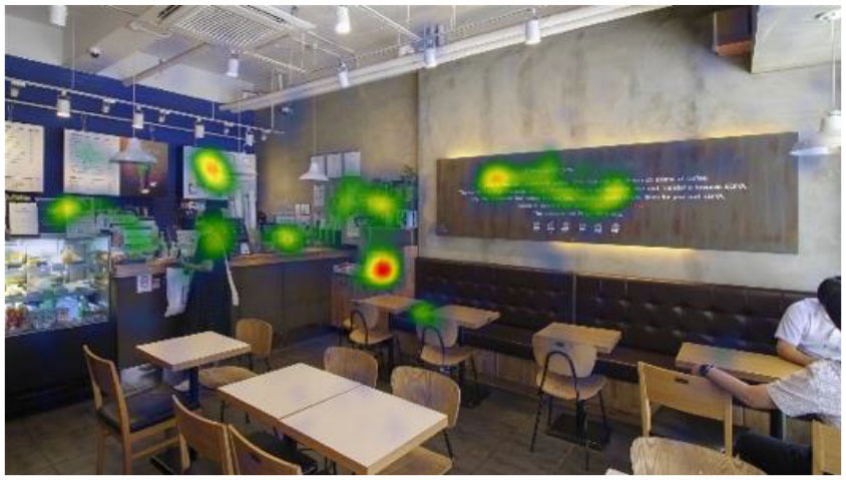

### Findings From Interviews: Meanings of Gaze Behaviors

The open-ended interview responses revealed the participants' personal experiences, thoughts, and feelings associated with their experience of the audio-visual stimuli, coffee shop interior images paired with musical pieces. Six essential meanings of their gaze behaviors were found through content analysis: (1) cross-sensory association and sensory overload, (2) attentional blindness, (3) the focal and ambient vision, (4) past experiences and meaning association, (5) attention to the unusual, and (6) human interaction and sociocultural association.

#### Cross-Sensory Association and Sensory Overload

While their wordings varied, most participants expressed cross-modal sensory associations to a certain degree, e.g., feeling (not seeing) visual crowdedness associated with the auditory stimuli and feeling (not hearing) noise in their visual perceptions. Participants also associated the brightness of the coffee shop images with noise or crowdedness. They felt the space in a photo they perceived brighter than the others busier and more crowded, despite the coffee shops in the three images were equally populated (Figure 1).

The coffee shop [I-1] felt a little…noisy. I could feel some motion…the people…I could tell that they were moving. I could almost hear a blender going on and off. I could see it getting crowded fast. … I saw two employees. (Participant #10—P10)

I felt like it [I-3] was quiet. And like I said, the grey color tones especially added to the quietness of the space. (Participant #03—P03)My eyes first went to the quote that was on the huge back-lit panel [I-3], which was like yelling out. (Participant #08—P08)

Other participants associated the photo images with a certain (imaginary) noise level, apart from the music played during the experiment. The reasons for the responses were varied. Despite the controlled sensory settings that excluded or minimized gustatory, olfactory, and tactile stimuli, several participants expressed sensory overload experienced when the auditory and visual information was provided during experiments. A few participants specifically mentioned that M2 in fast beats was obstructive while there was much visual information to comprehend:

When the faster music [M2] was playing, there was too much going on. I was trying to take in information and look around. Having extra stuff going on in the background, especially moving really fast and having a lot of musical parts to it, um…took away from the experience. It made it seem more crowded and less comfortable. (Participant #09—P09)

I feel like I generally leaned towards the Norah Jones song [M1] in…all of the spaces. The other song played, I think it was like a K-pop song or something. I don't know. It's kind of stuck in my head now. Yeah, I liked it. It just was a little too much. (Participant #18—P18)

Table 5 in the previous Section (0.1.3) showed the gaze plots associated with Participant #09 (P09) quoted above. With M2 (dance-pop) played, the fixations appeared more scattered, showing a lower fixation count per AOI than those with M1 (jazz-pop). Despite the pattern of fixations, P09 and several others recalled the visual features better while expressing their discomfort from the sensory overload. When participants said, “the music fit well with the space” or “the place seemed comfortable,” they tended to remember more of the overall ambiance than specific features. Four participants specifically mentioned their awareness of a conscious process in which they “noticed themselves” looking at things and feeling differently about the space when the music changed. The interview responses suggest that individuals' gaze- or fixation-patterns in eye-tracking do not always represent positive feedback or definition of the settings. Significantly short dwell-times and scattered fixations may reflect negative feedback rather than the positive in certain circumstances, e.g., perceiving space (vs. sales products) in commercial environments.

Uncertainty of task seemed to affect participants' feeling of sensory overload. With no specific task given to the participants other than looking at the images, they later expressed the minor stress felt during their eye-tracking experiments, i.e., the stress associated with “figuring out” where they were “supposed to look” while the background music was playing.

#### Attentional Blindness

Research has found that attentional blindness occurs during a high-pressure task, such as visual observation of an image when searching for a specific element or wayfinding in a spatial setting. Attentional blindness refers to “any failure to notice visual stimuli that can be attributed to attentional factors rather than perceptual impairment” (American Psychological Association, 2018). Although a high-pressure task becomes a stressor and results in attentional blindness in specific contexts, the current study found that attentional blindness can also occur in positive perceptual experiences. For example, P21 remembered many details of I-2, including the lighting fixtures and the ceiling color. However, the participant did not notice the exposed ceiling structure with a large suspended panel:

I liked it [I-2] was inviting and comfortable. I liked the long counter with light and the three big pendant lights over the long wood table. I don't remember the ceiling. I think it was dark—remember it was black. It had to be. I don't think that there was a lower ceiling—that also might have been exposed, but I just didn't notice it. (Participant #21—P21)

#### The Focal and Ambient Vision

Participants' interview responses revealed the association between their perceptions of objects and scenes and the focal and ambient vision. Studies have found that a built environment is experienced by two fundamental means: conscious focal processing and intellectual assessment and pre-conscious ambient spatial processing. While the central vision aids in processing focal awareness or conscious attention to objects, the peripheral vision aids in processing actions relating to objects and navigating motion through space (Rooney et al., 2017). In this study, the participants who mentioned the interior lighting of the coffee shop images associated it with the interior colors and materials first and then the overall atmospheres that are spatial contexts rather than objects or structural configurations on which one's gaze can focus. Thus, the participants' fixations and scanpaths could not provide evidence of meaningful attention to lighting even though they, including P12, specifically talked about their feelings associated with the interior lighting and color.

It was very obnoxious. The lighting [in I-2] was just so yellow, like a sickly kind of yellow, not a warm yellow. Just like uh… Just nasty. In your face, yellow. (Participant #12—P12)

Participants also expressed a sense of spatial volume, which is ambient awareness, associated with the impression of “cheap” or “fancy,” even though the three images used were of similar types of franchised brand stores with similar sizes. However, the participants' judgment did not always seem to reflect the participants' personal preferences.

The first one [I-1], it seemed really tight. That one to me, it felt almost kind of cheap at first. I think that also had to do with relation of places I've been to in the past. It had like a similar feeling so—it also after I compared it to the other two. (Participant #10—P10)

One [I-2] was the really big wooden one that had the bigger tables. It was the fanciest one. Uh, there were two women at the counter. Then the second one [I-3] was the smaller, cheaper one. I also liked the cheaper one was cozier. … I think it had like the white walls that just had the lights like these hanging down. And that one seemed like a lot more of a neighborhood coffee shop that I would go to. (Participant #12—P12)

The interview responses showed evidence of two different areas of visual perception. First, the central vision identifies an object and its visual properties (e.g., color, form, and material) perceived in high resolution. Second, peripheral vision relates to ambient awareness (e.g., detecting light, motion, and space). The results also suggest that the perception of light is one of the environmental factors that are less affected by attentional blindness than other visual objects or factors are. The interaction between light and color can be perceived regardless of particular gaze fixations, creating a general context, i.e., a kind of background. Due to the mechanism of the fovea, the degree of acuity in visual perception decreases depending on the distance from the visual attention point of the person, e.g., in the order of fine detail, gross detail, color, motion, and contrast and spatial volume.

#### Past Experiences and Meaning Association

Participants' perception of the audio-visual context used in this study appeared mainly as “preferred” or “fit in the context”; e.g., they “liked” or “disliked” the music, the music “went well with the space,” and the music “felt right in the space.” Specific association between the sound and the visual reflected their judgment based on their past experiences; e.g., the music makes the coffee shops look “high-end” or “low-key,” or the music fits a particular age group. While visual imagery directly influences what we see, recollections of past experiences may also shape perception itself (Pearson et al., 2008). Based on their personal experiences, the participants described the M1 as cliché, classic, nostalgic, moody, or natural and associated it with “high-end” interiors; alternatively, they described M2 as inviting, energy-boosting, vibrant, or upbeat, and associated it with “low-key” Interiors. However, such labeling did not provide a direct indication of personal preference.

They were playing Norah Jones music. It was…friendly, relaxing. The music added a lot to the atmosphere. My Mom had that on CD… kind of nostalgic to me. (Participant #13—P13)

Some of the stuff that I saw in the cheaper one looked like stuff I could get at IKEA. I kind of liked it. Now I feel bad to keep saying “cheap one.” Um…it's homely, let's call it that. (Participant #12—P12)

As these results suggest, gaze movement and fixation may not be direct indicators of individuals' preferences for their surroundings. Thus, using the gaze data without corresponding data such as interviews or surveys could result in misinterpretation of visual behavior.

#### Attention to the “Unusual”

Participants seemed to have certain expectations, e.g., where to look to find necessary information for their tasks or spatial characteristics with which they were familiar. They pointed out certain features that they found unusual or unexpected.

[I-1] Maybe it was a TV, but that wouldn't make a lot of sense for it to be a TV. It was like a woman who had a clipboard or something, and it was like a green background. Um… I don't know, it was super weird. I just kept looking at it. (Participant #19—P19)

A few participants who had worked at coffee shops responded from the point of view of an employee by envisioning themselves as working in the coffee shops they viewed.

That one [I-1] had like a lot of the extra stuff that I thought was unnecessary like they had the wooden bar fence coming up in the middle of the floor just to separate the people in line buying from the customers sitting down. So, I thought that was kind of useless and takes up space for no reason. I saw that and was like that just looks annoying. I used to have to sweep the floors and stuff, and you have to move tables and everything. So when I saw stuff that was heavy to move or stuff that was not moveable and was just in the middle of it I was just like uh. (Participant #10—P10)

So, [in I-1,] I saw the door to what I assume was the dish room or the stocking and stuff. Was away from the bar. It wasn't behind the counter. It's usually away from customers, and behind the counter, so they can't go in there and mess around. But it was right in the hallway where you can pass and go to the bathroom. That got on my nerves because you have to keep going in and out, and I wouldn't want to have to carry all of that stuff. I actually worked at Starbucks for years. So, when I looked at, it was like I was walking in to go to work. (Participant #12—P12)

Others pointed out a few other features that they felt stood out. The gaze plots and heatmaps (Tables 5, 6) shown in 4.1.3 also reflect their attention.

I liked their little quote on the wall [I-3]. Their business had like– I don't know- they were talking to their customers or whatever. (Participant #03—P03)

[I-3] My eye kept going back the blue background, painted brick, which I liked, and I felt like less corporate other than the sign with the quote. The menu…it seemed more handpicked where everything was than like I said, a template. (Participant #14—P14)

#### Human Interaction and Sociocultural Association

As mentioned previously, the gaze data showed a considerable pattern of fixations on human figures in the photo images, which were also reflected in the gaze plots (Table 5). Seven of 19 participants remembered the human interactions—either apparent or implied—they found in the images during their experiments.

There were definitely more people there [I-3], but I think that actually helps with making it more inviting. Like seeing other people there for some reason makes me think, “oh, I want to be here too. (Participant #21—P21)

I think there [I-3] were white tables, and then there were the two guys sitting in the corner. That felt like anyone could go into really—like you could just go with your friends. In the coffee shop [I-1] with the espresso table, there was like an older guy there, and he was on his phone. It just didn't really fit the scene, you know. (Participant #13—P13)

Individuals' sociocultural experience and understanding may play an important role in their spatial perception and interpretation. For example, several participants, including P03 and P13, associated particular visual elements with business aspects in their understanding.

I liked their little quote on the wall [I-3]. Their business had like– I don't know- they were talking to their customers or whatever. Their business was like that. (Participant #03—P03)

I know it [I-2] was really upscale because the lady that was ordering had like a Chanel bag—so I was like, “oh okay, its automatically upscale” because that's like thousands of dollars for the bag right there. So that kind of like changed my perspective. (Participant #13)

Individuals' familiarity or unfamiliarity with other types of sensory stimuli may also play an essential role in their visual perception and interpretation. Three participants associated M1 with high-class and M2 with low-key. They recognized the foreign language of the lyrics of the M2 and stated the coffee shop felt “cheap” because of the music. It was unclear whether the individuals' cultural or ethnic biases, rather than specific experiences, played a role in their responses.

When it was playing the calm, classical music [M1], the café [I-1] looked more rich and more high class. When they were like playing Korean pop songs, um, playing Korean pop songs [M2] made the café look a little bit cheaper, but more geared towards the younger audience like college kids or high school kids. But um, when it was playing the other song [M1], the Norah Jones, it seemed more formal. (Participant #14—P14)

In this statement, the participant was referring to the same image, making the two opposing judgments associated with the two auditory stimuli. A few participants also noticed the lyrics in a “foreign” language and found it distracting because they attempted to determine which language it was. The result suggests that unfamiliar auditory input was “additional information” for the participants to comprehend, and thus, it became a stressor or distraction.

## Discussion

This experimental phenomenological study investigated the impact of auditory stimuli on individuals' gaze behaviors and the hidden meanings of their audio-visual perceptions of commercial interiors. The participants' gaze behaviors were affected by auditory stimuli and other interior elements and factors associated with personal experiences; however, no distinct gaze pattern was identified by the type of auditory stimuli. Six essential meanings of their gaze behaviors were found through interviews: (1) cross-sensory association and sensory overload, (2) attentional blindness, (3) the focal and ambient vision, (4) past experiences and meaning association, (5) attention to the unusual, and (6) human interaction and sociocultural association.

While participants' gaze data revealed the visual elements at which the participants looked, the elements were not always remembered. This study also found that first gaze fixation, fixation count, and dwell time indicate or imply visual interests and distractors or stressors. These findings can explain the reason behind the finding by van Der Laan et al. (2015) that there is no significant or direct effect of the first fixation on consumer choice. Participants noticed the details of various interior elements such as finishes and materials, fixtures and furniture, and colors and lighting, as much as the spatial configurations. They remembered those factors as interior atmospheres or general styles rather than individual elements that constituted specific objects or architectonic space. They related the interplay among those factors, especially between color and light, to their feelings, preferences, and sensory associations. It suggests that visual attention to certain interior factors such as color, light, materials, and finishes cannot be adequately explained or measured by eye-tracking as a stand-alone; thus, designers must consider sound- or noise-reflection and absorption in planning interior materials and finishes, concerning the impact of auditory conditions on occupant perception of visual information.

Although studies argued that visual symmetry and directional configurations affect gaze fixations and sightlines (Treder, 2010; Atalay and Meloy, 2011; Goldberg and Helfman, 2011; Hodgson, 2011; Giannouli, 2013; Deng et al., 2016), this study showed no evidence of greater attention to visual symmetry nor directional patterns (e.g., horizontal vs. vertical sightlines) in the scanpaths and heatmaps of the gaze data. Such gaze patterns may occur in certain types of retail stores with many sales products, displays, and other related visual information but may be less relevant to other interior environments that involve many human actions and interactions in consumers' sight.

A considerable number of participants' first fixations appeared on human figures in the coffee shop images, and these revealed participants' interpretation of perceived human interactions. This result supports studies that proved the impact of the social relevance of sensory cues on visual attention and perception of space: customers have positive perception and feel more aroused or pleased in a store with social cues (Hu and Jasper, 2006); visual attention and spatial orienting are interpersonally attuned to the *social relevance* of the cues (Gobel et al., 2018). Thus, interior configurations and features that can promote human interaction can help create positive visual rhetoric contributing to the place identity and enrich customer experiences in commercial environments.

The impact of the sound properties of the musical stimuli on fixation count and time to the first fixation appeared dependent upon the individual participants' experiences. Instead, it appeared that the total amount of sensory stimuli as a whole had a greater impact on their visual experience and comfort levels. Any unfamiliar stimuli may affect one's sensory experience and become stressors or distractors. Several participants felt the Korean lyrics of one of the auditory stimuli distracting while looking at the coffee shop images; a few participants even mentioned it made the space look cheap. This suggests that when research tools or materials include culturally oriented components, it is important for the researchers to be aware that unfamiliar cultural factors can trigger one's discomfort or bias in spatial perception and its interpretation and judgment of the design. It is an important implication for interior architecture and design in which there has long been criticism that, despite the industry being globalized, the profession lacks diversity and related data (Nieminen, 2020).

Participants' gaze behaviors and visual preferences did not always correspond. Depending on the individual, great visual attention may represent either positive or negative interests or preferences. Interviews revealed the close relationship between the participants' personal preferences of the visual settings and their past experiences, many of which involved interaction with or judgment against other people. Thus, research methods to investigate visual attention and preference need researchers' further attention to avoid confusion or misinterpretation of the relationship.

There were limitations in this study. Individuals' gazes respond to three-dimensional spatial settings differently from two-dimensional still images. Using photo images lacking depth or distance projection might have affected participants' visual experience due to the limited range of perceptual and spatial variations in the on-the-screen spaces. In the same vein, the lab setting, including a screen-mounted eye tracker, could not sufficiently resemble a real-life space that involves various sensory factors, movement, and human interactions in its lived context. However, the lab-based eye-tracking experiments have meaningful implications for future studies involving various virtual environments that have increasingly become part of our everyday life. Several factors in this study might have added unintended distractions to the multisensory context, including the auditory stimuli with the lyrics in two different languages (English and Korean). The participants, English-first-language speakers, felt Korean, a foreign language to them, was distracting, perhaps because it involved another dimension of perception.

When asked during interviews, participants expressed that the silent 10-s break time between 60-s segments felt long and awkward because they were sitting in a dark room staring at a blank white screen. Although the speaker volume was set consistent during experiments, several participants reported they felt one auditory stimulus louder than the other, which informed that the sound decibels also need to be controlled to minimize confounding factors. Another factor to consider differently in future research design is the auditory stimuli repeated three times during each experiment, as the participant learned the pattern, possibly feeling sensory fatigue or losing interest.

## Conclusion

Adopting an experimental phenomenological approach, this study investigated the relationship between gaze behaviors, audio-visual stimuli in commercial interior environments, and the hidden meanings of the gaze behaviors. The findings suggest that auditory and visual stimuli are reciprocal in individuals' perceptions. Rather than one affects the other, the interaction between sensory stimuli contributes to the complexity and intensity of multisensory stimuli people associate with their experiences and conceptualize with meanings they establish. Although limitations were found, whether expected, most meaningful was the methodological exploration in this study that informed better about the environmental attributes affecting spatial perception and why they affect. Based on the thematic meanings found through this exploratory study, future studies may be designed with more controlled variables to scrutinize the specifics and their statistical significance.

Eye-tracking research in design disciplines has often focused on measuring gaze responses to identify visual elements that catch people's eyes. However, interior design researchers need to understand that eye-tracking is useful in measuring eye movement within the focal vision and gaze fixations on objects, but not for the spatial perception that involves certain attributes to which the peripheral vision responds, such as light, color ambiance, and spatial volume. This study shows that eye-tracking is not sufficient as a stand-alone method for interior design research on occupant visual behavior, especially for individuals' thoughts and feelings associated with their visual responses. Unless adopted for specific purposes, the traditional eye-tracking methods using two-dimensional images and screen-mounted eye trackers have unavoidable limitations in interior design research. Options for research on spatial perception can vary, e.g., screen-mounted vs.wearable eye tracker, still images vs.motion pictures, and multisensory stimuli vs.visual stimuli only. Varying viewing differences can also be considered, as increasing viewing distances can increase performance differences across eye-tracking devices (MacInnes et al., 2018). Because no one tool or material is always better than the others, determining an optimal combination of tools and materials can be the key to successful research.

Commercial interior environments continuously evolve as people engage in the ever-changing context. Designers desire to rigorously communicate with clients and occupants/users to learn from them, how they perceive their surroundings, and associate the settings with themselves. Design practitioners must understand that arbitrary assumptions or generalizations of certain interior elements' causal effects may be reliable. Since the hidden dimension of visual attention, perception, and preference has seldom been investigated, we hope that this study is the enterprise that opens a discussion that can contribute to the discovery of the deeper meaning of multifaceted occupant experience.

## Data Availability Statement

The datasets presented in this article are not readily available because the datasets are not to be released unless permitted by the funding and sponsoring institutions. Requests to access the datasets should be directed to Jain Kwon, jain.kwon@colostate.edu; Ju Yeon Kim, kjy@ssu.ac.kr.

## Ethics Statement

The studies involving human participants were reviewed and approved by the Institutional Review Board, Soongsil University. The patients/participants provided their written informed consent to participate in this study.

## Author Contributions

JK and JYK co-developed the study and conducted data collection and analysis. JK interpreted the data and wrote the paper. The authors provided final approval of the version submitted for review and publication.

## Conflict of Interest

The authors declare that the research was conducted in the absence of any commercial or financial relationships that could be construed as a potential conflict of interest.

## Publisher's Note

All claims expressed in this article are solely those of the authors and do not necessarily represent those of their affiliated organizations, or those of the publisher, the editors and the reviewers. Any product that may be evaluated in this article, or claim that may be made by its manufacturer, is not guaranteed or endorsed by the publisher.
